# Retirement’s impact on health: what role does social network play?

**DOI:** 10.1007/s10433-023-00759-w

**Published:** 2023-05-10

**Authors:** Asal Pilehvari, Wen You, Xu Lin

**Affiliations:** 1 Department of Public Health Sciences, University of Virginia, Charlottesville, USA; 2 Department of Economics, Virginia Tech, Blacksburg, USA

**Keywords:** Retirement, Physical and mental health, Social network, Mediation, I12, J26

## Abstract

While a large body of literature investigates the bidirectional relationship between retirement and health, few have analyzed the mechanism through which retirement affects health which will provide important policy instrument insights. Using three waves of National Social Life, Health, and Aging Project, we examine the mediating role of the social network in the relationship between retirement and health in USA. We address the endogeneity and reverse causality through panel instrumental fixed-effect methods. We apply both single and parallel mediation analyses to identify the potential mechanism by which social network characteristics mediate the impact of retirement on health. Findings reveal that retirement adversely affects physical and mental health outcomes, and a considerable portion of these effects are explained by social network changes post-retirement. Specifically, 58% of reduction in the probability of reporting good physical health and 4.5% of increment in chances of having depression symptoms post-retirement can be explained by shrinkage in the size of social network in retirees. Using parallel mediation identification to account for dependencies among social network features, we find that social network size induces 79.5% reduction in probability of reporting good physical health and 18.6% increase in probability of having depression in retirees as compared to non-retirees. Findings in this paper suggest that investing in social network of the elderly can buffer the adverse health effect of retirement and can be an effective policy target for promoting healthy aging.

## Introduction

In the USA, population aging so-called silver tsunami causes deep social and political transformations, challenging society in many aspects. The continuing reduction in ratio of workers to retirees causes serious concerns about Social Security benefit sustainability, and growing of elderly population due to improvement in longevity increases the burden on medical care and pension systems. In particular, Social Security paid out more benefits than it collected in taxes in 2018, and recent prediction by Social Security Administration shows the trust will be depleted by 2034.^1^

These concerns prompt a series of policies such as increasing the Social Security retirement age and Medicare eligibility age. The effectiveness of increasing retirement age depends on an implicit assumption that late retirement is good for health or at least does not harm health. Retirement is a life-changing event that can improve or deteriorate elderly’s health both physically and mentally (Nishimura et al. 2018; Dave et al. 2008; Coe and Lindeboom 2008). Therefore, the precise identification of the causality impacts of retirement on the elderly’s health is needed for effective policy design, implementation, and evaluation.

However, establishing the causal effects of retirement on health is empirically challenging for a couple of reasons. First, existing evidence suggests a reverse causal relationship between health and labor supply decisions and second, the existence of unobserved confounding factors that influence both health and retirement decisions simultaneously such as work environment and genetics.

Furthermore, as a life-changing event, retirement significantly alters retirees’ daily routines, social contact, and social activities (i.e., Barnett et al. 2012; Eibich 2015). If retirement affects social network while social network significantly impacts individual’s health (Cohen 2004), social network might be a health policy instrument that can be used to promote elderly’s health, i.e., minimizing the negative impact of post-retirement’s life changes on retiree’s health through maintaining and fostering retirees’ social capital.

However, disentangling the causality between health and social network suffers similar identification problem because the formation of social network is not random (Manski 1993; Moffitt et al. 2001; Brock and Durlauf 2001): individuals’ unobserved heterogeneity simultaneously affects both health production and social network formation. People who have lower discount rates and put more value for future benefits are more likely to invest in health and social network that results in better health and more social capital in the future. Meanwhile, healthy individuals have more energy and time to socialize with others and enrich their social network. As a result, a model that examines the causality of retirement and social network on retirees’ health outcomes will need to address the endogeneity problem that may cause inconsistent estimations.

The literature has applied various identification methods to address the aforementioned empirical challenges in investigating the relationship between retirement and health but has not reached consistent findings (e.g., Kofi Charles 2004; Neuman 2008; Coe and Lindeboom 2008; Coe and Zamarro 2011; Gorry et al. 2018; Insler 2014). The most recent studies (e.g., Gorry et al. 2018; Insler 2014) used instrumental-variable (IV) and fixed-effect (FE) methods with early and regular pension benefit eligible ages as instruments to deal with the endogeneity in the retirement status.

It is still hard to compare results across studies since they use different retirement definitions such as not working for pay and working less than 1200 h per year. Those definitions do not necessarily capture the effect of complete retirement (i.e., not working at all both for pay and for free) on health precisely.^2^ Furthermore, to the best of our knowledge, there is no empirical evidence on the social network pathway through which retirement affects health.

The chief goal of this study is to identify the mediatory impact of social network on the subsequent health effect of retirement using the egocentric social network and health information available in the National Social Life, Health and Aging Project (NSHAP) survey (discussed in detail in later section). For this purpose, we first investigate the retirement impact on health and address the endogeneity issue by employing the panel structure of NSHAP.^3^ In particular, we tackle the endogeneity caused by unobservables and individual’s heterogeneity by using eligibility age for full Social Security benefits as an instrumental variable for retirement status as well as applying fixed-effect methodology. Furthermore, we restrict our sample to pre-retirement healthy individuals to minimize the potential inconsistency induced by reverse causality and potential weak instrument problem (i.e., people might retire in other time in the life span, not necessarily at the eligibility age for Social Security benefits). Lastly, we highlight the importance of investigating the causal relationship between retirement and health and the mediatory role of social network for public policy intervention to promote healthy aging.

## Review of literature

### Health on retirement

On top of the numerous factors that affect retirement decision, such as Social Security eligibility, financial resources, and health insurance, health is believed to be a crucial determinant. The empirical findings are consistent in supporting this conclusion. By modeling the endogenous health dynamics within a structural model of retirement, Bound et al. (2010) find that healthy people are unlikely to retire unless they have a sizable financial resource. It is more likely for those who are in poor health to retire before being eligible for any pension benefits. Capatina (2015) indicates four channels by which health affects an individual’s labor supply: productivity, medical expenditures, available time, and mortality. She states that productivity and time lost to sickness are the main channels by which health affects labor supply. Similarly, Gustman and Steinmeier (2018) demonstrate that improving the overall health of the population would delay retirement by one year. Moreover, McGarry (2004) shows that the impact of changes in health on retirement expectations is much greater than financial variables.

### Retirement on health

Recently, researchers have paid more attention to the effect of retirement on health outcomes. Several studies show a significant health improvement after retirement (e.g., Charles 2002; Bound and Waidmann 2007; Johnston and Lee 2009; Neuman 2008; Coe and Lindeboom 2008; Coe and Zamarro 2011; Insler 2014), while others find that retirement significantly deteriorates health (e.g., Dave et al. 2008; Behncke 2012). Recently, a systematic review (Nishimura et al. 2018) points out that different methodology utilized is a key factor for explaining the mixed results in the literature as well as the choice of wide ranges of control variables. For example, in a study using UK data, Behncke (2012) employs a nonparametric matching approach and finds no impact of retirement on depression along with a negative impact on self-reported health. However, by using Regression Discontinuity Design, Johnston and Lee (2009) indicate that retirement lowers depression for a sample of men who do not have an educational degree in UK.^4^ Besides, different norms, labor market, and economic incentives embedded in the Social Security and pension system across different countries may also contribute to the inconsistency in empirical findings in the literature.

Even if we only compare the studies on US data, the findings are still mixed. For example, in terms of subjective well-being, by using Social Security normal retirement age as an instrumental variable, Kofi Charles (2004) finds that retirement has a positive effect on subjective well-being, while Dave et al. (2008) find no effect. For the self-reported health, most findings show that the probability of reporting good health increases after retirement (e.g., Neuman 2008; Coe and Lindeboom 2008; Calvo et al. 2011; Nishimura et al. 2018), whereas some reports the opposite (Dave et al. 2008). In the case of physical health, Dave et al. (2008) report retirees are more likely to suffer from difficulties in their physical activities, while other studies declare there is a positive association between retirement and physical health for women but none for men (Neuman 2008; Nishimura et al. 2018). In regard to mental health, some studies claim that retirement does not affect depression (Neuman 2008; Coe and Lindeboom 2008), while others find that retirement is associated with higher depression (Nishimura et al. 2018; Li et al. 2021).

### The role of social network

A few studies investigate how retirement changes the social life and social network of retirees (e.g., Comi et al. 2020; Patacchini and Engelhardt 2016; Börsch-Supan and Schuth 2014; Barnett et al. 2012). Social network is defined as a “web of social relationships surrounding an individual and the characteristics of those ties” (Berkman et al. 2000), which are generally characterized in terms of structure, quality, and function. Social network structure refers to the number of people in the network and the level of contact an individual has with the other network members.

An important function of a social network is to provide social support, especially emotional and instrumental support, to members of the network that eventually influence mental and physical health (especially for those older individuals) (e.g., Israel 1982; Cohen 2004; Ronconi et al. 2012; Petrou and Kupek 2008; Fiori and Jager 2012; Litwin and Shiovitz-Ezra 2010; Allen et al. 2014). Social network members can provide social support and companionship for day-to-day adaptation to new life of retirees that will contribute to better health. According to Berkman and Syme (1979), individuals who have larger social networks are more likely to have better health, especially when the network members are frequently contacting each other (Terhell et al. 2007). Social network involvement might include negative social interaction in which social network members behave in hurtful and inconsiderate ways that result in worse health (Krause and Rook 2003). Noticeably, studies found that positive social interactions and social support happen much more frequently than do negative interactions (Rook 1998).

In a recent study on 11 European countries, Comi et al. (2020) report major changes in the structure of the social network upon retirement. They conclude retirees make stronger ties with family members, while they lose ties with friends and colleagues. In a similar study on the same countries, Börsch-Supan and Schuth (2014) find early retirement reduces the size and intensity of relationships in retiree’s social network, which leads to significant early cognitive aging. In the US study, researchers Patacchini and Engelhardt (2016) find similar results on the negative impact of retirement on size and density of social network.

The effect of retirement on social network is an empirical hypothesis. On the one hand, retirement reduces social interactions because of losing co-workers and work-related networks and thus shrinks the size of social network (Sugisawa et al. 1997). On the other hand, people would have more time to do voluntary works, participate in different types of social activities, and make new connections to expand their social networks (Barnett et al. 2012).^5^ No matter which direction the impact is, retirement will lead to social network changes and subsequently may impact retirees’ health. Therefore, it is needed to investigate the empirical evidence of social network’s mediation effect in the pathway between retirement and health to understand whether social network is a viable channel to invest on for healthy aging promoting policies.

## Data and variables

### Data

In our empirical analysis, we use data from the National Social Life, Health, and Aging Project (NSHAP), which is conducted by National Opinion Research Center (NORC) at the University of Chicago. This is a population-based panel study of the US elderly with specific purpose to investigate connections between health and social factors. The first wave of the NSHAP includes a sample of 3,005 adults aged 57–85 years old (born between 1920 and 1947) who were interviewed in 2005 or 2006. Wave 2 consists of 2261 Wave 1 respondents who were re-interviewed in 2010 or 2011. Wave 2 also includes the cohabiting spouses and romantic partners of Wave 1 respondents in addition to Wave 1 non-interviewed respondents. In total, wave 2 includes 3400 respondents. Wave 3 constitutes of 4777 individuals who were interviewed in wave 2 in addition to a new cohort born between 1948 and 1965 (baby boomers).^6^ The construction of the subsample used in our study will be explained in Sect. 3.5.

### Health variables

As outcome variables, we consider both physical and mental health measures available in NSHAP. Physical health: We examine the overall physical health based on the respondent’s answer to the question “would you say your physical health is excellent, very good, good, fair, or poor?” The response is coded on scale from 1 to 5. Higher values of self-reported physical health correspond to better health. The physical health outcome in the analysis is an indicator variable equal to one if respondent reports excellent or very good health status (Coe and Lindeboom 2008).

*Depression* The NSHAP includes a depression scale introduced by Center for Epidemiologic Studies (CES), which is based on a cumulative summation over response scores to eleven questions. Respondents were asked about the frequency of certain feelings in the past week (e.g., “how often did you feel depressed in the past week?”). Certain feelings include: Depressed, Restless, Difficult, Poor appetite, Everything was an effort, Happy, Lonely, People were unfriendly, Enjoyed life, Sad, and Being disliked. There are four possible responses: “rarely or none of the time” (score: 1); “some of the time” (score: 2); “occasionally” (score: 3); and “most of the time” (score: 4).^7^Higher values of CESD represent more depression symptoms and worse mental health. A score of 16 is considered as a standard cutoff point (Radloff 1977), meaning that scores greater than 16 denote the existence of depression symptoms. Using this cutoff point, we define a dichotomous variable for depression in which value one indicates the existence of depression symptoms, and zero otherwise.

*Anxiety* NSHAP includes seven questions related to anxiety symptoms defined by Hospital Anxiety and Depression Scale (HADS). Respondents were asked about the frequency of feeling anxiety symptoms (i.e., tense, something awful about to happen, restless, worried, relaxed, frightened, and panic) in the past week. The range of answer to these questions are similar to the CES-D depression. Hence, we calculate the anxiety score by summation over the response scores to the seven questions. The higher values of anxiety variable demonstrate worse mental health.^8^ Score 8 is considered as cutoff point such that scores below 8 show no symptoms of Anxiety (Zigmond and Snaith 1983). Anxiety variable in our analysis is an indicator variable with value one indicating presence of anxiety symptoms.

### Social network variables

NSHAP includes the respondent’s egocentric social network. An egocentric social network includes an ego (the respondent) and a set of members. In NSHAP, respondents could name up to five people that immediately surround them in the past 12 months, but respondents were also asked to denote if they had more than five members in their networks. Also, NSHAP contains frequency of contacts among members including the respondent. Frequency of contacts are collected by asking respondents “how often do you talk to the person cited?” The responses range “have never spoken to each other (0),” “less than once a year (1),” “once a year (2),” “a couple of times a year (3),” “once a month (4),” “once every two weeks (5),” “once a week (6),” “several times a week (7),” “every day (8).” In NSHAP, respondents are asked to describe type of their relationship with each member in the network (e.g., partner, family, friend, coworker, etc.).

Based on available information about social network in NSHAP, we construct the most frequent examined network characteristics in the social network literature: size of social network, frequency of contacts, and diversity of ego’s network (Carolan 2013). Size is defined as number of members in the respondent’s social network. We construct an index for measuring frequency of contacts by summation over the scores of ego’s frequency of contacts (i.e., scores range from 0 to 8) with members and normalize it with the size of network. This normalization makes the frequency measure less dependent on the size. To illustrate this, consider ego1 who has one member in her social network and every day is in contact with that person as compared to ego2 who has 8 members in his network and is in contact with them less than a day per year. In terms of the frequency of contacts, without normalization, these two have the same frequency of contacts (i.e., 8).

Based on varieties of relationship types in NSHAP, we define 8 categories: partner, parent, child, family, friend, neighbor, coworker, and others (i.e., minister, priest, or other clergy, psychiatrist, psychologist, counselor, or therapist, caseworker/social worker, and house-keeper/home healthcare provider). We utilize the Index of Qualitative Variation (IQV) (Knoke and Yang 2008) to construct the diversity measurement of social network. For the i-th respondent with N members in the network, where members are classified into K categories, the IQV is defined as follows:

1 $$\begin{aligned} \text {IQV}=\frac{1-\Sigma _{j=1}^{k}P_j^2}{\frac{k-1}{k}} \end{aligned}$$

In which $P_j$ is the percentage of members of network in the j-th category. The IQV is a standardized measure ranging between 0 and 1, where 0 indicates all N members are in one category and 1 indicates members are equally dispersed across K categories.

### Retirement and control variables

NSHAP contains demographic and socioeconomic information such as gender, age, ethnicity, marital status, educational attainment, income, and employment status (i.e., currently working, retired, disabled, unemployed, homemaker, or other). Dichotomous indicator is defined to be 1 for retirement if the respondent reports retired and not working, the indicator is 0 otherwise.^9^ We focus on full retirement to capture the largest effect that retirement can have on health. The NSHAP also includes respondent’s health behaviors like smoking and drinking. Particularly, respondents were asked whether they smoke cigarettes currently and how many cigarettes they smoke per day, whether they currently drink alcohol and the number of drinks they consume per day. In our empirical analysis, we control for these observable characteristics (e.g., age, income, marital status, cigarette smoking, and alcohol consumption).^10^

### Sample selection

We restrict the sample to subsample of healthy individuals before retirement event. Although we lose many observations, we immune our findings from potential simultaneity problem between retirement and health. There are several advantages for us to focus on pre-retirement healthy individuals: first, it is less likely that health causes retirement for these individuals (i.e., reverse causality is minimized). Second, the inconsistency induced by unobservable confounding factors that simultaneously influence both health and retirement is also minimized. Third, this sample selection strategy also protects our estimations from weak instrument inconsistency (more details will be provided in “Empirical methodology” section).

Likewise, this subsample selection helps minimize the inconsistency caused by the possible endogeneity of social network as well. Remarkably, social network involvement might not only be disturbed by health problems, but also be changed by retirement. Focusing on the subsample of healthy individuals before retirement guarantees bad health does not interfere the social network changes. In particular, individuals are defined as healthy if they report good, very good, or excellent as their self-reported physical and have depression and anxiety score less than 16 and 8, respectively.^11^ Consequently, our pre-retirement healthy subsample includes 1,160 individuals. Hence, we sacrifice the variation in observations for the sake of validity of results and preventing inconsistency in the estimations.

### Summary statistics

Table 1 provides data description and summary statistics of the variables in the selected subsample of pre-retirement healthy individuals and compares retirees versus non-retirees. According to Table 1, 45% of sample are retired who are on average significantly older than non-retirees (i.e., 70 years old vs. 66 years old).^12^ The mean of self-reported physical health depicts that on average retired people are in worse health condition as compared to non-retirees. Table 1 also indicates retirees on average experience higher levels of depression and anxiety symptoms than non-retirees do.

**Table 1:** Summary statistics and data description of subsample of study

	Not-retired	Retired	Difference	Min^a^	Max^b^
*Employment status*					
Retirement	0	1	− 1	0	1
*Health outcomes*					
Physical health	3.89	3.51	0.38***	1	5
	(0.76)	(1.00)	(0.08)		
Depression	12.88	13.93	− 1.05***	11	29
	(1.96)	(3.16)	(0.23)		
Anxiety	2.39	3.48	− 1.08***	0	14
	(2.23)	(3.18)	(0.25)		
*Demographics*					
Age	66.24	69.95	− 3.70***	57	90
	(6.01)	(6.02)	(0.61)		
Female	0.46	0.44	0.02	0	1
	(0.50)	(0.50)	(0.05)		
Married	0.73	0.67	0.06	0	1
	(0.44)	(0.47)	(0.05)		
White	0.85	0.83	0.02	0	1
	(0.36)	(0.38)	(0.04)		
Asian	0.05	0.05	− 0.01	0	1
	(0.21)	(0.23)	(0.02)		
High education	0.75	0.74	0	0	1
	(0.44)	(0.44)	(0.04)		
High income	0.88	0.81	0.06*	0	1
	(0.33)	(0.39)	(0.03)		
*Health behaviors*					
Alcohol consumption	0.69	0.59	0.09**	0	1
	(0.46)	(0.49)	(0.05)		
Number of drinks	1.3	1.09	0.22	0	15
	(1.51)	(1.16)	(0.15)		
Smoking cigarette	0.11	0.09	0.02	0	1
	(0.31)	(0.28)	(0.03)		
*Social network*					
Size^c^	9.46	9.52	− 0.05	2	18
	(3.08)	(3.18)	(0.31)		
Frequency of contacts^d^	14.85	15.63	− 0.79	2	31.3
	(5.15)	(5.65)	(0.53)		
Diversity^e^	0.67	0.64	0.03	0	0.95
	(0.19)	(0.21)	(0.02)		
Observations	633	537			

This table reports the summary statistics of the subsample of healthy (healthy in self-reported physical and mental health, no depression, and no anxiety) individuals before retirement. Retired refers to individuals who reported retired and not working

^a^Min denotes the minimum values of each variable

^b^ Max denotes the maximum values of each variable

^c^Size refers to the number of members in the individual’s social network

^d^Frequency refers to the frequency of contacts with members of one’s social network

^e^Diversity refers to diversity in the types of relationships in individual’s social network that is measured by Index of Qualitative Variation (IQV). Standard deviations are in parentheses. For some variables, the actual sample size is less due to missing information and because some of the variables are in the leave-behind questioners

Asterisks present that the difference between the retired and non-retired samples is statistically significant as follow: *** significant at the 1% level; ** significant at the 5% level; * significant at the 10% level

According to this table, on average, retirees have lower income as compared to non-retirees. With regard to health behaviors, retirees drink alcohol less than non-retirees on average, whereas no significant difference exists between these two groups in terms of smoking. While retirees on average seem to have larger social network with higher frequency of contacts and lower diversity in their network as compared to non-retirees’ social network, no significant differences exist on these characteristics among the two groups.

Some limitations in the data set prevent us from a comprehensive investigation of the effects of social network. In particular, we have no information about the geographical distance between respondents and members, the form of communication between social network members whether it is by mail, internet, in person, or by phone. Also, the lack of detailed information on health condition and labor status of the social network members deprives the opportunity for examination of potential interactive and spillover social effects. Moreover, although health insurance is a key variable in late life that influences both health and retirement decision, we exclude it from our empirical analysis due to the lack of variations in the sample. Particularly, more than 80% of the sample have health insurance in the first two waves.

## Empirical methodology

### Retirement and health

For simplicity, consider the following linear specification of health as a function of retirement in Eq. (2):

2 $$\begin{aligned} H_{it}= \alpha _0+ cR_{it}+\alpha _2 X_{it}+\mu _i+\epsilon _{it} \end{aligned}$$

3 $$\begin{aligned} R_{it} = \delta _0+ \delta Z_{it}+\delta _2 X_{it}+\mu _i+\nu _{it} \end{aligned}$$

where $H_{it}$ is the health status of individual *i* at time *t* and $R_{it}$ is retirement status of individual *i* at time *t*. $X_{it}$ denotes time-variant observable characteristics such as age, income, marital status, and health behaviors. $\mu _i$ indicates time-invariant unobservable characteristics of individuals such as genetics, family background, and time preferences. $\epsilon _{it}$ and $\nu _{it}$ are i.i.d error terms. We are interested in consistent estimation of *c*, which is a challenging task due to reasons discussed earlier, i.e., the reverse causal relationship between health and labor supply decision, and the existence of unobserved confounding factors that influence both health and retirement decision simultaneously.

To account for endogeneity in retirement (Angrist and Keueger 1991; Cameron and Trivedi 2005), we apply Instrumental Variables (IV) strategy by using eligibility age for full entitlement Social Security benefits (i.e., 65 years old; $Z_{it}=1(\text {Age}\ge 65)$) as instrument (see Appendix for more details and the first-stage estimation results).

In addition, addressing the heterogeneity effect is also essential. Due to different job characteristics and socioeconomic background, some individuals may experience better health after retirement, whereas others may experience no changes or deterioration on health upon retirement. Besides, heterogeneity in health investment behaviors might be another source of variations in health after retirement (Grossman 1972). According to Grossman’s model, health is both investment and consumption goods. Upon retirement, with no incentive to invest in health to increase productivity and thus earnings, individuals may not sufficiently invest in their health, which lead to poor health post-retirement. However, health as a consumption good directly enters the utility function and retirees may invest more in it, which results in better health post-retirement.

Failing to consider these individual heterogeneity leads to inconsistent estimation of retirement’s impact on health. We exploit the panel nature of data and apply individual fixed-effects (FE) method that controls for all unobserved time-invariant heterogeneity across individuals (Wooldridge 2010; Cameron and Trivedi 2005). Although using IV can help to minimize the endogeneity issue, full retirement age for eligibility of Social Security benefit might be a weak instrument because individuals retire all the time during the life span (i.e., even after full retirement age).^13^ To avoid the inconsistency of estimations, we restrict the sample to pre-retirement healthy individuals. For healthy individuals before retirement, retirement is less likely to be endogenous. Therefore, we capture the causality of retirement on subsequent health by applying the FE-IV approach in Eqs. (2) and (3) to the subsample of healthy individuals before retirement.

### Mediation effect of social network

**Fig. 1:**
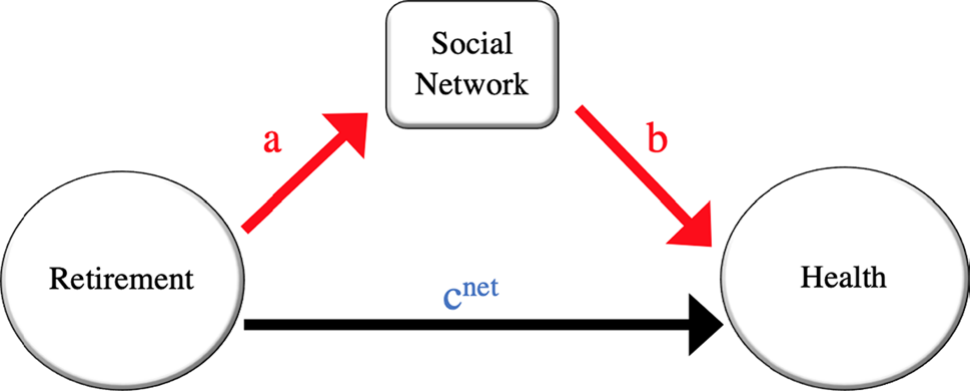
Single mediation model of social network on the relationship between retirement and health

We are particularly interested in the mediation effect of social network on post-retirement changes in health. According to Fig. 1, two pathways exist for retirement to influence health. One is the direct effect, which refers to the pathway from retirement to health without passing through social network. The other one is the indirect effect, which refers to the pathway from retirement to health through social network. If retirement alters the social network characteristics (e.g., lost the contact with colleagues or raising opportunity to engage in different social activities), and social network impacts individual’s health, then social network would be a health policy instrument that can be used for intervention in promoting elderly’s health.

The corresponding econometric model is as follows^14^:

4 $$\begin{aligned} \text {SNW}_{it} = & \alpha _{0}+{a}R_{it}+\alpha _{1} X_{it}+\mu _{i}+\nu _{it}\nonumber \\ H_{it} = & \beta _0+{{c}^{\text {net}}} R_{it}+{b}\text {SNW}_{it}+\beta _1 X_{it}+\mu _{i}+\epsilon _{it} \end{aligned}$$

where $SNW_{it}$ refers to a specific social network characteristic (i.e., size, frequency of contacts, or diversity), and the rest of the variables are as defined previously. $\nu _{it}$ and $\epsilon _{it}$ present the i.i.d error term in each equation, respectively.

According to Baron and Kenny (1986), the multiplication of a and b is the social network mediation on the health impact of retirement, and $c^{net}$ is the direct impact of retirement on health holding social network variable constant. Therefore, the summation of $c^{net}$ and $a \times b$ indicates the total effect of retirement on subsequent health. To tackle the endogeneity problem, we apply the FE-IV approach to each equation using the subsample of pre-retirement healthy individuals.

**Fig. 2:**
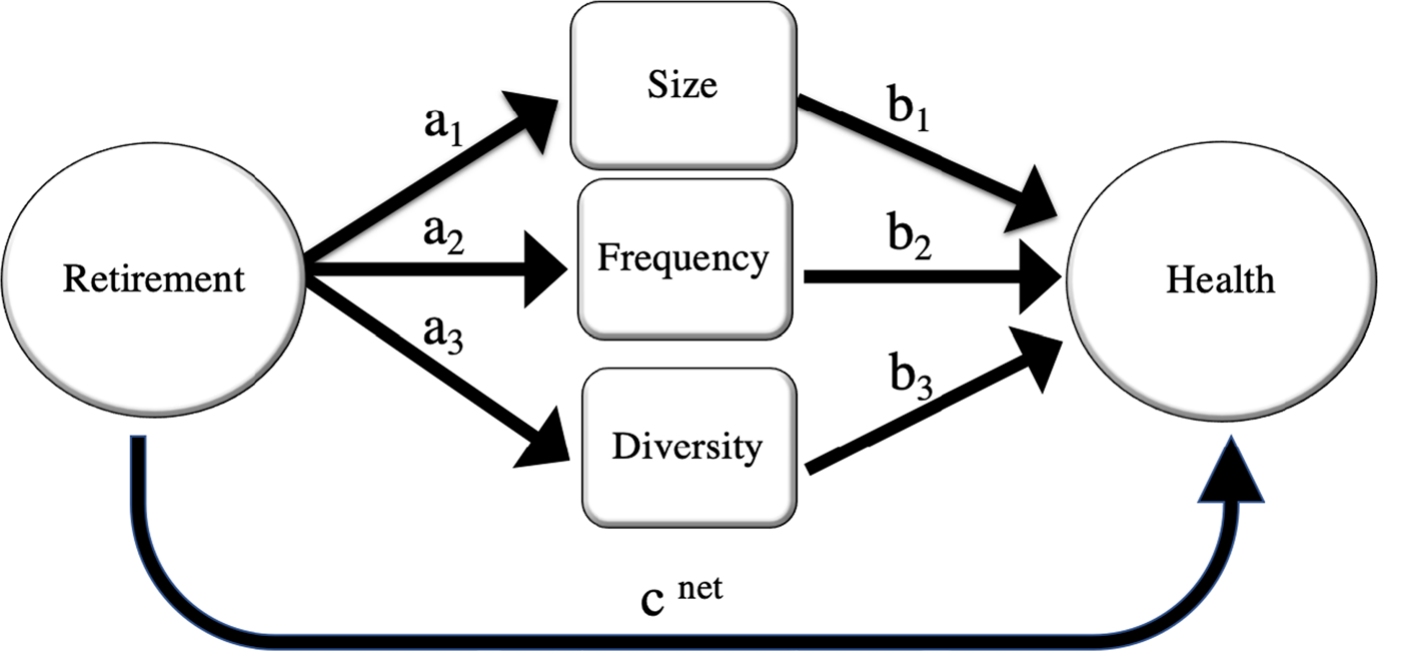
Parallel mediation model of social network on the relationship between retirement and health

From a policy perspective, it would be interesting to know if any of the social network characteristics drives the mediation more than the others. Parallel mediation analysis presented in Fig. 2 is a method to compare magnitudes of the indirect effects while allows for the correlations among the mediators. As shown in Fig. 2, the absence of any arrows linking the mediators (i.e., social network characteristics) assumes no causality link exists between them.

The corresponding econometric model is defined as follows:

5 $$\begin{aligned} \left[ {\begin{array}{*{20}c} {{\text{Size}}_{{it}} } \\ {{\text{Frequency}}_{{it}} } \\ {{\text{Diversity}}_{{it}} } \\ {H_{{it}} } \\ \end{array} } \right] & = \left[ {\begin{array}{*{20}c} {\beta _{0} } \\ {\beta _{1} } \\ {\beta _{2} } \\ {\beta _{3} } \\ \end{array} } \right] + \left[ {\begin{array}{*{20}c} {a_{0} } \\ {a_{1} } \\ {a_{2} } \\ {c^{{{\text{net}}}} } \\ \end{array} } \right]R_{{it}} + \left[ {\begin{array}{*{20}c} 0 & 0 & 0 \\ 0 & 0 & 0 \\ 0 & 0 & 0 \\ {b_{1} } & {b_{2} } & {b_{3} } \\ \end{array} } \right]\left[ {\begin{array}{*{20}c} {{\text{Size}}_{{it}} } \\ {{\text{Frequency}}_{{it}} } \\ {{\text{Diversity}}_{{it}} } \\ \end{array} } \right] \\ & \; + \left[ {\begin{array}{*{20}c} {\alpha _{0} } \\ {\alpha _{1} } \\ {\alpha _{2} } \\ {\alpha _{3} } \\ \end{array} } \right]{\mathbf{X}}_{{it}} + \left[ {\begin{array}{*{20}c} 1 \\ 1 \\ 1 \\ 1 \\ \end{array} } \right]\mu _{i} + \left[ {\begin{array}{*{20}c} {\nu _{{0it}} } \\ {\nu _{{1it}} } \\ {\nu _{{2it}} } \\ {\nu _{{3it}} } \\ \end{array} } \right] \\ \end{aligned}$$

or,

6 $$\begin{aligned} {\textbf{Y}}={\beta }+{\textbf{a}}R_{it}+{\textbf{B}}\text {SNW}_{it}+\mathbf {\alpha }\mathbf {X_{it}}+\mathbf {l_4}\mu _i+{\nu } \end{aligned}$$

where all variables are similar to those defined previously in Eqs. (3) and (2). ${\textrm{Size}}_{it}$ refers to social network size of individual *i* at time *t*, ${\textrm{Frequency}}_{it}$ indicates the frequency of contacts of individual *i* with members of her social network at time t, and ${\textrm{Diversity}}_{it}$ refers to diversity index (i.e., IQV) for an individual *i* at time *t*.

According to Fig. 2, we have three different indirect effects of retirement on health such that each one passes through one social network characteristic ($a_1 \times b_1$, $a_2 \times b_2$, and $a_3 \times b_3$). The sum of the three indirect effects and the direct effect of retirement gives the total effect of retirement on health. Similar to single mediation analysis, we implement the FE-IV approach to each equation on the subsample of pre-retirement healthy individuals to address endogeneity.^15^ All the analysis are done using STATA 14^16^(StataCorp 2015).

## Empirical results

### Total effect of retirement

The first set of analysis examines the total impact of full retirement on subsequent health. Table 2 presents the total effect of retirement on self-reported physical health, depression, and anxiety by FE-IV estimation of Eqs. (3) and (2) as the first stage.^17^ This evidence shows, in line with previous findings Dave et al. (2008), that retirement generates a significant adverse effect on physical health, depression, and anxiety in elderly. However, the primary focus of this study is to reveal how social network can mediate the effect of retirement on subsequent health.

**Table 2:** FE-IV estimation of total impact of retirement on subsequent health outcomes

	Physical health	Depression	Anxiety
Retirement	− 0.093*** (0.04)	0.220*** (0.04)	0.113*** (0.03)
Observations	1101	1098	1008

Each cell presents the total effect of retirement on corresponding health outcomes using the fixed-effect instrumental variable estimation method. In all analyses, we control for health behaviors, age, marital status, and income levels. Instrument is a dummy variable with value 1 if individual is older than 65 years old, and 0 otherwise. The sample is limited to pre-retirement healthy individuals in all these four health outcomes. The health outcomes are binary variables. Table 7 in Appendix reports the detailed estimation results. Robust individuals-clustered standard errors are in parenthesis. *** indicate the significant at the 1% level

### Single mediation analysis

Estimation results of the single mediation model (i.e., Fig. 1) are shown in Table 3. Each panel of the table provides results for a specific health outcome, and each row presents the coefficient estimation of equations in system (4) considering one specific social network feature as the mediator variable.

**Table 3:** Single mediation analysis: FE-IV estimation of mediatory impact of social network characteristic on retirement’s effect on physical health, depression, and anxiety

	Retirement impact on SNW	Social Network impact on Health	Mediation impact of SNW	Direct effect of retirement on health
	*a*	*b*	$a \times b$	$c^{\textrm{net}}$
*Physical health*				
Size^d^	− 4.032***	0.013**	− 0.054***	− 0.039
	(0.323)	(0.006)	(0.001)	(0.052)
Frequency^e^	− 2.392***	0.003	− 0.007***	− 0.085**
	(0.504)	(0.004)	(0.000)	(0.042)
Diversity^f^	− 0.035*	0.015	− 0.001***	− 0.092**
	(0.021)	(0.084)	(0.000)	(0.038)
Observations	1101			
*Depression*				
Size	− 4.044***	− 0.003	0.010***	0.211***
	(0.323)	(0.005)	(0.001)	(0.048)
Frequency	− 2.404***	0.002	− 0.005***	0.226***
	(0.504)	(0.004)	(0.000)	(0.038)
Diversity	− 0.036*	0.110	− 0.004***	0.225***
	(0.021)	(0.080)	(0.000)	(0.036)
Observations	1098			
*Anxiety*				
Size	− 4.247***	− 0.000	0.001*	0.111***
	(0.344)	(0.005)	(0.001)	(0.038)
Frequency	− 2.486***	0.000	− 0.001***	0.113***
	(0.503)	(0.003)	(0.000)	(0.028)
Diversity	− 0.044**	0.039	− 0.002***	0.114***
	(0.021)	(0.059)	(0.000)	(0.026)
Observations	1008			

Each row reports the fixed-effect Instrumental Variable estimation results of equations corresponding to Fig. 1

^*d*^Size refers to the number of members in the individual’s social network. ^*e*^Frequency refers to the frequency of contacts with members of one’s social network. ^*f*^Diversity refers to diversity in the types of relationships in individual’s social network that is measured by Index of Qualitative Variation (IQV). The sample is limited to pre-retirement healthy individuals in all these four health outcomes. Instrument is a dummy variable with value 1 if individual is older than 65 years old, and 0 otherwise. Tables 8, 9,  10 and in Appendix report the detailed estimation results. Robust individuals-clustered standard errors are in parenthesis. The significance level is defined as follows: *** significant at the 1% level; ** significant at the 5% level; * significant at the 10% level

We first explain how retirement affects social network features. In Table 3, “a” refers to the estimation of retirement’s impact on the size, frequency, and diversity of social network (corresponding to “a” in Fig. 1), separately. The result shows that retirement significantly reduces the size of social network, frequency of contacts (Sugisawa et al. 1997), and the diversity in one’s network.

The second column in the table reports the estimated impact of social network characteristics on the health outcomes—after controlling for the time-invariant effects. Larger social network is associated with higher probability of reporting good physical health, while has no impact on depression and anxiety, holding everything else constant. Frequency of contacts and diversity in network do not impact any of the health outcomes (i.e., *b* = 0), holding all else constant.^18^ Even if social network does not generate a significant direct impact on health, it could still perform as a mediator on the impact of retirement on health. As a matter of fact, according to a recent study Zhao et al. (2010), it is not necessary to have a significant a or b to establish a mediation effect; instead, the distribution of their products matters for the existence of mediation effect (also, Hayes 2017). We obtain the empirical distribution of $a \times b$ using bootstrapping to calculate the standard errors and find that it is significant at the 95% level.

The third column displays the mediatory effect of social network features on the path from retirement to health. As we see, the social network plays a significant mediatory role with the highest impact of size in comparison with the other two features. Remarkably, size shrinkage explains 58% (i.e., $\frac{0.054}{0.093}*100$) of reduction in probability of reporting good physical health caused by retirement, frequency of contacts explains around 7.5% of it, with a small mediatory contribution of diversity.

Depression: social network size reduction explains $\frac{0.010}{0.22}*100= 4.5\%$ of increment in probability of having depression symptoms upon retirement. However, the other two features of social network, the frequency of contacts and diversity, have a small mediatory impact on depression upon retirement. Thus, the size of retiree’s social network explains the highest adverse effect of retirement on depression as compared to the other two features.

Anxiety: social network size marginally increases the probability of having anxiety symptoms in retirees. Interestingly, findings show that reduction in frequency of contacts and diversity upon retirement significantly contribute to the mitigation of anxiety. This can be explained by the concept of social anxiety in elderly and negative impact of social network involvements that is discussed earlier. Social anxiety implies elderlies in contact with others often feel they are a burden on the life of people surrounding them. However, the impact of frequency of contacts and diversity on reducing probability of having anxiety symptoms upon retirement is small. The direct impact of retirement on anxiety dominates the mediatory effect of social network characteristics and leads to overall higher anxiety symptoms in retirees as compared to non-retirees. There might be other hidden factors that fuels the impact of retirement on anxiety such as financial fears and lifestyle changes.

The last column of Table 3 shows that the direct effect of retirement is significant on all health outcomes ($c^{\textrm{net}}\ne 0$) except physical health, while the mediator is social network size. The nonzero direct impact of retirement on health might be due to all the other mechanisms through which retirement might influence health such as financial hardships, health insurance coverage, and lifestyle changes.

In summary, simple mediation analysis suggests social network as a significant channel that influences various health outcomes upon retirement. In the next section, we consider the mediatory effects of all three social network features simultaneously in the model.

### Parallel mediation analysis

According to Fig. 2, retirement is modeled to exert its effect on health through 4 pathways. One pathway is direct, from retirement to health without passing through any of the proposed social network mediators, and the other three pathways are indirect, each through one feature of social network. The estimation results corresponding to Fig. 2 (i.e., system of equations in (5)) are shown in Table 4. These parallel analyses shed more light on the social network mechanism for the post-retirement decline in health.

**Table 4:** Parallel mediation analysis: FE-IV estimation of mediatory impact of social network characteristics on retirement’s effect on physical health, depression, and anxiety

Health outcomes	$c^{net}$	Mediation effects			Total indirect effect
		Size^a^	Frequency^b^	Diversity^c^	
		$a_1 \times b_1$	$a_2 \times b_2$	$a_3 \times b_3$	
Physical health	− 0.030***	− 0.074***	0.009***	0.003***	− 0.062***
	(0.002)	(0.001)	(0.000)	(0.000)	(0.001)
Depression	0.198***	0.041***	− 0.013***	− 0.006***	0.023***
	(0.002)	(0.001)	(0.000)	(0.000)	(0.001)
Anxiety	0.107***	0.009***	− 0.002***	− 0.002*** (0.000)	0.005***
	(0.001)	(0.001)	(0.000)		(0.001)

Each row reports the fixed-effect Instrumental Variable estimation results of equations corresponding to Fig. 2

^a^Size refers to the number of members in the individual’s social network

^b^ Frequency refers to the frequency of contacts with members of one’s social network

^c^ Diversity refers to diversity in the types of relationships in individual’s social network that is measured by Index of Qualitative Variation (IQV). The sample is limited to pre-retirement healthy individuals in all these four health outcomes. Instrument is a dummy variable with value 1 if individual is older than 65 years old, and 0 otherwise. Tables 11, 12, and 13 in Appendix report the detailed estimation results. Robust individuals-clustered standard errors are in parenthesis

*** indicates significant at the 1% level

In the first row of Table 4, we see that retirement significantly decreases probability of reporting good physical health, ceteris paribus—holding social network variables and all other explanatory variables constant. The mediation coefficient estimation of social network size implies that retirees are likely to report worse physical health than non-retirees due to a smaller social network size at the same levels of frequency of contacts and diversity. Particularly, size shrinkage explains 79.5% of reduction in the chance of reporting good physical health among retirees, while holding frequency of contacts and diversity constant. In contrast, retirees are estimated to have better physical health in comparison with non-retirees through the effect of retirement on the frequency of contacts/diversity, holding size, and everything else constant. In contrast to single mediation analysis, the parallel mediation analysis reflects that with the same social network size and diversity, higher frequency of contacts is associated with higher chance of reporting good physical health. It reflects the importance of considering the correlation between the social network features in the analysis rather than assessing them in isolation from the other related features. Overall, the adverse mediatory effect of size dominates the impact of the other two features of social network. Therefore, the indirect and direct impact of retirement reduces the probability of reporting good physical health by 6.2% and 3%, respectively.

As expected, retirees are more likely to experience depression symptoms by 19.8% than non-retirees. Overall, we observe similar patterns in direct and indirect effects of depression to physical health. In particular, about 18.6% increase in probability of having depression symptoms after retirement is explained by the reduction in the size of social network, holding frequency of contacts and diversity constant. Within the parallel mediation framework, the impact of retirement on depression through social network is more than 10%$\left(\frac{0.023}{0.22}*100= 10.5\%\right)$.

In parallel mediation analysis, smaller social network size is associated with higher chance of having anxiety symptoms post-retirement, holding the frequency of contacts and diversity of network constant. The size of social network contributes to higher chances of anxiety in retirees by 7.9% $\left(\frac{0.009}{0.113}*100= 7.9\%\right)$, whereas the frequency of contacts and diversity estimation show similar effects to single mediation analysis in the parallel system.

Comparing the mediatory effects of social network features across health outcomes, we find that size of social network generates the largest effect in comparison with the other two features for health outcomes. According to the last column of Table 4, retirement decreases the chance of reporting good physical health and increases the probability of having depression and anxiety symptoms through its influence on social network features. These results imply that adverse health impacts post-retirement can be curbed by improving social network of retirees.

## Discussion

Different conceptual theories in psychology and sociology address how social network influences health or vice versa (e.g., Israel 1982), while no study empirically investigates the effect of health on social network. Yet, health might be a potential factor affecting network formation since the formation of social network is not random (Moffitt et al. 2001; Brock and Durlauf 2001). For instance, healthier people are more likely to participate in social activities and have more social connections. Besides, individuals’ heterogeneity, such as time preferences and personality, might simultaneously contribute to the extent of social network involvement and health conditions. For example, extrovert people are more likely to participate in different social activities, meanwhile they are less likely to have depressive symptoms.

However, the severity of endogeneity between health and social network formation is less severe in older adults as compared to younger adults. According to socioemotional selectivity theory (SST) (Carstensen 1991), as people age, they focus on enriching and maintaining the existing relationships rather than investing in new ones. That is, the effect of unobservables on the older adults’ health is less likely to influence their social network formation, meaning that unobservable confounding factors are not significant matter of endogeneity between health and social network in this population. In addition, in this study, the social network and health are not measured at the same time. There is a temporal exogeneity between social network and health. Respondents were asked about their social network in the last 12 months, while the health-related questions were asked at the moment of the interview. Therefore, it is less likely that their current health influences the social network characteristics within the past year. As we limit the sample to healthy individuals before retirement event, we further assure that health does not interfere the changes in social network upon retirement as well (i.e., no reverse causality). However, we acknowledge that this study is limited by lack of information for developing a valid and relevant instrument for social network to sufficiently tackle the endogeneity of health and social network.

## Policy implications

Evidence provided by this study has important policy implications. The key message in this study is that considering the health challenges caused by population aging, social capital (in the form of social network) is a key policy instrument for health promotion in the elderly. Investing in the social capital of the elderly may help to curb the negative health effect post-retirement, even if the retirement eligibility age remains unchanged.

Enriching social network in elderly may also help to ameliorate the depression symptoms after retirement. Our finding indicates that a large portion (i.e., 4.5% in single mediation analysis and 18.6% in parallel mediation estimation) of increase in the chance of having post-retirement depression symptoms happens due to a reduction in social network size.

Therefore, interventions that target promoting different aspects of the elderly’s social capital buildup are promising ones to allocate resources into, aiming to improve elderly’s health. Particularly, the government can improve health of the elderly by investing in their social capital. For example, one effective policy is to provide education on technologies that can minimize social network size shrinkage in retirees. Also, the government can directly provide subsidies to promote the elderly’s social capital enrichment, for instance, by organizing community elderly activities targeting groups that share similar prior occupations, or by providing funding to stimulate community participation through voluntary organizations and community groups.

## Conclusions

In this study, we investigate how retirement can impact health through altering social network characteristics, including size, diversity, and frequency of contacts. First we estimate the health impact of retirement using the FE-IV method with Social Security benefits eligibility age as instrument for retirement on pre-retirement healthy individuals in NSHAP data set. Then, we investigate the mediatory role of social network on the health impact of retirement. We investigate the mediatory effect of social network characteristics in single and parallel mediation model specifications, separately. The parallel analysis has the advantage of accounting for possible dependence and correlation between different social network features, while single analysis considers one social network feature at a time.

Estimations reveal a statistically significant negative effect of retirement on physical health, depression, and anxiety. Findings uncover that retirees not only have fewer members in their social network but also have less frequent contacts with members as compared to non-retirees. Due to the association of social network and health, differences in social network of retirees and non-retirees explain a substantial amount of disparities in health outcomes between the two groups. Our findings indicate that a considerable portion of retirement’s impact on health is mediated by social network changes.

In particular, single mediation identification suggests that 58% of reduction in the chances of being in good physical health and 4.5% of increment in the probability of having depression post-retirement can be explained by the reduction in social network size, whereas, in the parallel identification, holding the other social network features constant, the corresponding portions of the effects of social network size reduction on negative physical health outcome and depression upon retirement become 79.5% and 18.6%, respectively. We find similar impact of social network size reduction on chances of having anxiety with lower magnitude of the effect. Moreover, with the same social network size, retirees who have higher frequency of contacts/diversity experience lower chances of anxiety, which can be explained by social anxiety concept in elderly.

We find social network changes due to important lifestyle change after retirement and can be utilized as an effective policy instrument to buffer the adverse health outcomes of retirement. Social network based interventions that target social capital buildup in older adults might be desirable for healthy aging. For instance, the government can provide subsidies for organizing and establishing different community events to stimulate social capital buildup for the elderly. Understanding the underlying mechanisms of how social network can mediate the adverse effect of retirement on health calls for more detailed information about the structure of social network of the elderly. This will be investigated in a future project as more social network data become available.

There are some limitations to this study. First, the exact retirement time is not identifiable since the survey wave time gap is five years. Therefore, we assume respondents retired within the past five years (if they reported retired status in the current wave and not reported as retired in the previous wave). Second, this study is limited by a lack of information for developing a valid and relevant instrument for the social network to sufficiently tackle the endogeneity of health and social network. Third, we restrict the sample to pre-retirement healthy individuals to address the potential endogeneity problem. Therefore, our findings might likely be conservative in the effect magnitude since our analysis does not quantify the effects of retirement on those who are not healthy before retirement. At last, the NSHAP dataset includes no information on health and labor status of social network members. Therefore, we could not examine the potential interactive and spillover social effects.

## Figures and Tables

**Fig. 3:**
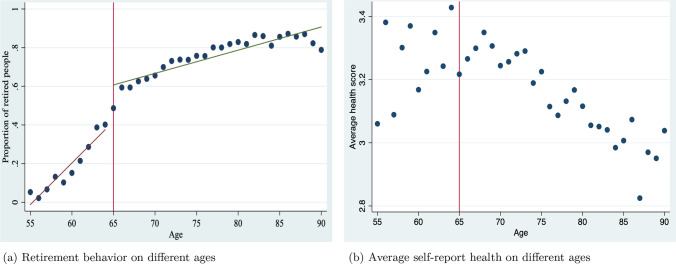
Retirement and health behavior of different ages. Higher health score means better self-report health

## Appendix

### Instrumental variable method

To be a valid instrument, an instrument must be correlated to retirement (endogenous regressor) and related to health outcomes only through the effect on retirement (i.e., corr $(Z_{it},\epsilon _{it})=0$). Well-documented literature exists about the sharp changes in retirement behavior around this age (e.g., Coile and Gruber 2000; Ruhm 1995), which confirms this cutoff point should have the power to predict retirement decision. It is worth mentioning that an individual’s health outcomes do not change substantially by officially turning one year older, even though health gradually declines as people aged.

Empirical findings using NSHAP also provide evidence for the discrete changes in retirement decision around the eligibility age for full Social Security benefits. Figure 3a presents the jump in retirement behavior at 65 years old, while Fig. 3b confirms the self-reported health does not necessarily decrease around this point. Therefore, eligibility age for full Social Security benefits is a valid and relevant instrument. Fixed-effect regression estimation of equation (3) as the first stage shows that reaching 65 years old is significantly associated with a 34-percentage-point increase in retirement for the pre-retirement healthy individuals subsample. Table 5 presents the first-stage estimation results.

**Table 5:** First-stage results of FE-IV estimations

	Dep var: Retirement			
	Physical health	Mental health	Depression	Anxiety
Instrument (full Social Security entitlement age indicator)^a^	0.342***	0.345***	0.346***	0.333***
	(0.04)	(0.04)	(0.04)	(0.05)
Age	0.011***	0.010***	0.011***	0.010***
	(0.00)	(0.00)	(0.00)	(0.00)
Income	0.006	0.008	0.008	0.016
	(0.01)	(0.01)	(0.01)	(0.02)
Married^b^	0.009	0.004	0.006	− 0.007
	(0.03)	(0.03)	(0.03)	(0.03)
Number of drinks	− 0.003	− 0.003	− 0.004	− 0.006
	(0.01)	(0.01)	(0.01)	(0.01)
Smoking cigarette	− 0.033	− 0.025	− 0.046	− 0.033
	(0.05)	(0.05)	(0.05)	(0.05)
Constant	− 0.568***	− 0.523***	− 0.563***	− 0.529**
	(0.19)	(0.20)	(0.19)	(0.21)
Observations	1101	1080	1098	1008
F_statistics	60.27	57.19	61.65	52.14

This table reports the first stage results of FE-IV estimation of retirement on health outcomes in subsample of healthy individuals before retirement

Robust individuals-clustered standard errors are in parenthesis

^a^The indicator is 1 if the individuals is 65 years old or older, and 0 otherwise

^b^ Married is a dummy variable that takes value of 1 if individual is married, and 0 otherwise. The sample is limited to pre-retirement healthy individuals in all these four health outcomes

*** indicates significant at the 1% level

See Tables 6, 7, 8, 9, 10, 11, 12 and 13.

**Table 6:** Ordinary Least Square estimation of impact of social network characteristics on health outcomes

	Physical health	Depression	Anxiety
Size^a^	0.012**	− 0.014***	− 0.005
	(0.006)	(0.004)	(0.003)
Frequency^b^	− 0.009**	0.004	0.001
	(0.004)	(0.003)	(0.002)
Diversity^c^	− 0.053	0.113**	0.029
	(0.079)	(0.057)	(0.045)
Age	− − 0.004*	0.008***	0.004***
	(0.002)	(0.002)	(0.001)
Female	0.052	0.043*	0.045**
	(0.032)	(0.023)	(0.018)
High education^d^	0.076**	− 0.018	− 0.032
	(0.035)	(0.025)	(0.020)
Income	0.039**	− 0.015	0.006
	(0.017)	(0.012)	(0.010)
Married^e^	0.061	− 0.045*	0.039*
	(0.038)	(0.027)	(0.021)
Number of drinks	0.030***	− 0.007	− 0.002
	(0.011)	(0.008)	(0.006)
Smoking cigarette	0.008	0.019	0.010
	(0.051)	(0.036)	(0.028)
Constant	0.731***	− 0.396***	− 0.250**
	(0.191)	(0.136)	(0.108)
Observation	1101	1098	1008

This table reports the OLS estimation results of social network characteristics on different health outcomes

Robust individuals-clustered standard errors are in parenthesis

^a^Size refers to the number of members in the individual’s social network

^b^Frequency refers to the frequency of contacts with members of one’s social network

^c^Diversity refers to diversity in the types of relationships in individual’s social network that is measured by Index of Qualitative Variation (IQV)

^d^Education levels equal to some college or higher is considered as high education

^e^Married is a dummy variable that takes value of 1 if individual is married, and 0 otherwise. The sample is limited to pre-retirement healthy individuals in all these four health outcomes

The significance level is defined as follows: *** significant at the 1% level; ** significant at the 5% level; * significant at the 10% level

**Table 7:** FE-IV estimation of total impact of retirement on health outcomes

	Physical health	Depression	Anxiety
Retirement^a^	− 0.093**	0.220***	0.113***
	(0.04)	(0.04)	(0.03)
Age	− 0.087*	0.064	0.059*
	(0.05)	(0.04)	(0.03)
Income	− 0.014	0.014	0.037**
	(0.02)	(0.02)	(0.02)
Married^b^	0.103	− 0.263***	− 0.060
	(0.07)	(0.07)	(0.06)
Number of drinks	0.035**	− 0.022*	0.002
	(0.01)	(0.01)	(0.01)
Smoking cigarette	0.069	− 0.141*	− 0.083
	(0.10)	(0.08)	(0.09)
Constant	0.549***	0.241***	− 0.006
	(0.08)	(0.08)	(0.06)
Observations	1101	1098	1008

This table reports the fixed-effect Instrumental Variable estimation of retirement on health outcomes. Instrument is a dummy variable with value 1 if individual is older than 65 years old, and 0 otherwise

^a^Retirement is 1 if the individual reports retired and not working, and 0 otherwise

^b^Married is a dummy variable that takes value of 1 if individual is married, and 0 otherwise. The sample is limited to pre-retirement healthy individuals in all these four health outcomes. Robust individuals-clustered standard errors are in parenthesis

The significance level is defined as follows: *** significant at the 1% level; ** significant at the 5% level; * significant at the 10% level

**Table 8:** Single mediation analysis: FE-IV estimation of mediatory impact of social network characteristic on retirement’s effect on physical health

	Size^a^	Physical health	Frequency^b^	Physical health	Diversity^c^	Physical health
Retirement	− 4.032***	− 0.039	− 2.392***	− 0.085*	− 0.035	− 0.092**
	(0.391)	(0.060)	(0.484)	(0.048)	(0.022)	(0.045)
Age	− 0.868**	− 0.082	− 0.700	− 0.085	0.005	− 0.086
	(0.381)	(0.057)	(0.506)	(0.055)	(0.022)	(0.056)
Income	0.349*	− 0.019	0.265	− 0.015	0.006	− 0.014
	(0.210)	(0.024)	(0.260)	(0.024)	(0.012)	(0.024)
Married^d^	0.806	0.092	3.439***	0.092	0.092***	0.101
	(0.602)	(0.069)	(0.744)	(0.070)	(0.034)	(0.070)
Number of drinks	0.191	0.033**	0.173	0.035**	0.000	0.035**
	(0.138)	(0.016)	(0.170)	(0.016)	(0.008)	(0.016)
Smoking cigarette	− 0.210	0.075	− 0.168	0.073	0.095**	0.071
	(0.820)	(0.094)	(1.014)	(0.094)	(0.046)	(0.095)
Size		0.013**				
		(0.006)				
Frequency				0.003		
				(0.004)		
Diversity						0.015
						(0.083)
Constant	7.877***	0.443***	11.678***	0.513***	0.568***	0.540***
	(0.740)	(0.108)	(0.914)	(0.104)	(0.041)	(0.098)
Observations	1101	1101	1101	1101	1101	1101

This table presents the fixed-effect Instrumental Variable estimation results of single mediation equations corresponding to Fig. 1. Instrument is a dummy variable with value 1 if individual is older than 65 years old, and 0 otherwise. For instance, column 2 presents the estimation result of retirement on the size of social network and column 3 presents the estimation of retirement and size of social network on physical health

^a^ Size refers to the number of members in the individual’s social network

^b^ Frequency refers to the frequency of contacts with members of one’s social network

^c^ Diversity refers to diversity in the types of relationships in individual’s social network that is measured by Index of Qualitative Variation (IQV)

^d^ Married is a dummy variable that takes value of 1 if individual is married, and 0 otherwise. The sample is limited to pre-retirement healthy individuals in all health outcomes in this study. Robust individuals-clustered standard errors are in parenthesis

The significance level is defined as follows: *** significant at the 1% level; ** significant at the 5% level; * significant at the 10% level

**Table 9:** Single mediation analysis: FE-IV estimation of mediatory impact of social network characteristic on retirement’s effect on Depression

	Size^a^	Depression	Frequency^b^	Depression	Diversity^c^	Depression
Retirement	− 4.044***	0.211***	− 2.404***	0.226***	− 0.036*	0.225***
	(0.392)	(0.052)	(0.485)	(0.043)	(0.022)	(0.040)
Age	− 0.873**	0.064	− 0.701	0.062	0.004	0.060
	(0.378)	(0.045)	(0.503)	(0.043)	(0.021)	(0.043)
Income	0.318	0.014	0.198	0.013	0.005	0.013
	(0.211)	(0.021)	(0.261)	(0.021)	(0.012)	(0.021)
Married^d^	0.833	− 0.261***	3.623***	− 0.271***	0.085**	− 0.273***
	(0.612)	(0.061)	(0.757)	(0.062)	(0.034)	(0.062)
Number of drinks	0.177	− 0.023	0.173	− 0.023*	− 0.001	− 0.023
	(0.139)	(0.014)	(0.172)	(0.014)	(0.008)	(0.014)
Smoking cigarette	− 0.203	− 0.118	− 0.174	− 0.117	0.097**	− 0.128
	(0.823)	(0.083)	(1.018)	(0.083)	(0.046)	(0.083)
Size		− 0.003				
		(0.005)				
Frequency				0.002		
				(0.004)		
Diversity						0.110
						(0.073)
Constant	7.952***	0.260***	11.721***	0.214**	0.579***	0.176**
	(0.743)	(0.095)	(0.919)	(0.091)	(0.041)	(0.086)
Observations	1098	1098	1098	1098	1098	1098

This table presents the fixed-effect Instrumental Variable estimation results of single mediation equations corresponding to Fig. 1. Instrument is a dummy variable with value 1 if individual is older than 65 years old, and 0 otherwise. For instance, column 2 presents the estimation result of retirement on the size of social network and column 3 presents the estimation of retirement and size of social network on depression

^a^ Size refers to the number of members in the individual’s social network

^b^ Frequency refers to the frequency of contacts with members of one’s social network

^c^ Diversity refers to diversity in the types of relationships in individual’s social network that is measured by Index of Qualitative Variation (IQV)

^d^ Married is a dummy variable that takes value of 1 if individual is married, and 0 otherwise. The sample is limited to pre-retirement healthy individuals in all health outcomes in this study. Robust individuals-clustered standard errors are in parenthesis

The significance level is defined as follows: *** significant at the 1% level; ** significant at the 5% level; * significant at the 10% level

**Table 10:** Single mediation analysis: FE-IV estimation of mediatory impact of social network characteristic on retirement’s effect on anxiety

	Size^a^	Anxiety		Frequency^b^	Anxiety		Diversity^c^	Anxiety
Retirement	− 4.247***	0.111***		− 2.486***	0.113***		− 0.044**	0.114***
	(0.408)	(0.043)		(0.497)	(0.034)		(0.022)	(0.032)
Age	− 0.528*	0.064*		− 0.407	0.061*		0.007	0.059*
	(0.316)	(0.037)		(0.435)	(0.036)		(0.020)	(0.036)
Income	0.387*	0.037**		0.158	0.037**		0.007	0.037**
	(0.220)	(0.017)		(0.269)	(0.017)		(0.012)	(0.017)
Married^d^	0.799	− 0.060		2.988***	− 0.061		0.116***	− 0.065
	(0.650)	(0.051)		(0.793)	(0.051)		(0.035)	(0.051)
Number of drinks	0.103	0.002		0.095	0.002		− 0.005	0.002
	(0.145)	(0.011)		(0.177)	(0.011)		(0.008)	(0.011)
Smoking cigarette	− 0.142	− 0.083		− 0.249	− 0.083		0.091*	− 0.086
	(0.863)	(0.068)		(1.052)	(0.068)		(0.047)	(0.068)
Size		− 0.000						
		(0.005)						
Frequency					0.000			
					(0.003)			
Diversity								0.039
								(0.063)
Constant	7.916***	− 0.004		12.287***	− 0.010		0.560***	− 0.028
	(0.773)	(0.077)		(0.943)	(0.075)		(0.042)	(0.071)
Observations	1008	1008		1008	1008		1008	1008

This table presents the fixed-effect Instrumental Variable estimation results of single mediation equations corresponding to Fig. 1. Instrument is a dummy variable with value 1 if individual is older than 65 years old, and 0 otherwise. For instance, column 2 presents the estimation result of retirement on the size of social network and column 3 presents the estimation of retirement and size of social network on anxiety

Robust individuals-clustered standard errors are in parenthesis

^a^Size refers to the number of members in the individual’s social network

^b^ Frequency refers to the frequency of contacts with members of one’s social network

^c^ Diversity refers to diversity in the types of relationships in individual’s social network that is measured by Index of Qualitative Variation (IQV)

^d^ Married is a dummy variable that takes value of 1 if individual is married, and 0 otherwise. The sample is limited to pre-retirement healthy individuals in all health outcomes in this study

The significance level is defined as follows: *** significant at the 1% level; ** significant at the 5% level; * significant at the 10% level

**Table 11:** Parallel mediation analysis: FE-IV estimation of mediatory impact of social network characteristics on retirement’s effect on physical health

	Size^a^	Frequency^b^	Diversity^c^	Physical Health
Retirement	− 4.032***	− 2.392***	− 0.035	− 0.030**
	(0.391)	(0.484)	(0.022)	(0.003)
Size				0.018**
				(0.008)
Frequency				− 0.004
				(0.005)
Diversity				− 0.083
				(0.091)
Age	− 0.868**	− 0.700	0.005	− 0.080
	(0.381)	(0.506)	(0.022)	(0.056)
Income	0.349*	0.265	0.006	− 0.019
	(0.210)	(0.260)	(0.012)	(0.024)
Married^d^	0.806	3.439***	0.092***	0.109
	(0.602)	(0.744)	(0.034)	(0.071)
Number of drinks	0.191	0.173	0.000	0.032**
	(0.138)	(0.170)	(0.008)	(0.016)
Smoking cigarette	− 0.210	− 0.168	0.095**	0.083
	(0.820)	(1.014)	(0.046)	(0.095)
Constant	7.877***	11.678***	0.568***	0.498***
	(0.740)	(0.914)	(0.041)	(0.112)
Observations	1101	1101	1101	1101

Note: This table presents the fixed-effect Instrumental Variable estimation results of parallel mediation equations corresponding to Fig. 2 with physical health as the outcome variable. Instrument is a dummy variable with value 1 if individual is older than 65 years old, and 0 otherwise. Columns 2–4 present the FE-IV estimation of social network variables on the retirement, corresponding to the paths in Fig. 2

Robust individuals-clustered standard errors are in parenthesis

^a^Size refers to the number of members in the individual’s social network

^b^Frequency refers to the frequency of contacts with members of one’s social network

^c^Diversity refers to diversity in the types of relationships in individual’s social network that is measured by Index of Qualitative Variation (IQV)

^d^Married is a dummy variable that takes value of 1 if individual is married, and 0 otherwise. The sample is limited to pre-retirement healthy individuals in all health outcomes in this study

The significance level is defined as follows: *** significant at the 1% level; ** significant at the 5% level; * significant at the 10% level

**Table 12:** Parallel mediation analysis: FE-IV estimation of mediatory impact of social network characteristics on retirement’s effect on depression

	Size $^a$	Frequency$^b$	Diversity$^c$	Depression
Retirement	− 4.044***	− 2.404***	− 0.036*	0.200***
	(0.392)	(0.485)	(0.022)	(0.003)
Size				− 0.010
				(0.007)
Frequency				0.005
				(0.004)
Diversity				0.152*
				(0.080)
Age	− 0.873**	− 0.701	0.004	0.060
	(0.378)	(0.503)	(0.021)	(0.044)
Income	0.318	0.198	0.005	0.015
	(0.211)	(0.261)	(0.012)	(0.021)
Married$^d$	0.833	3.623***	0.085**	− 0.287***
	(0.612)	(0.757)	(0.034)	(0.063)
Number of drinks	0.177	0.173	− 0.001	− 0.022
	(0.139)	(0.172)	(0.008)	(0.014)
Smoking cigarette	− 0.203	− 0.174	0.097**	− 0.134
	(0.823)	(1.018)	(0.046)	(0.083)
Constant	7.952***	11.721***	0.579***	0.168*
Observations	1098	1098	1098	1098

This table presents the fixed-effect Instrumental Variable estimation results of parallel mediation equations corresponding to Fig. 2 with depression as the outcome variable. Instrument is a dummy variable with value 1 if individual is older than 65 years old, and 0 otherwise. Columns 2-4 present the FE-IV estimation of social network variables on the retirement, corresponding to the paths in Fig. 2

Robust individuals-clustered standard errors are in parenthesis

^a^ Size refers to the number of members in the individual’s social network

^b^ Frequency refers to the frequency of contacts with members of one’s social network

^c^ Diversity refers to diversity in the types of relationships in individual’s social network that is measured by Index of Qualitative Variation (IQV)

^d^ Married is a dummy variable that takes value of 1 if individual is married, and 0 otherwise. The sample is limited to pre-retirement healthy individuals in all health outcomes in this study

The significance level is defined as follows: *** significant at the 1% level; ** significant at the 5% level; * significant at the 10% level

**Table 13:** Parallel mediation analysis: FE-IV estimation of mediatory impact of social network characteristics on retirement’s effect on anxiety

	Size^a^	Frequency^b^	Diversity^c^	Anxiety
Retirement	− 4.247***	− 2.486***	− 0.044**	0.107**
	(0.408)	(0.497)	(0.022)	(0.004)
Size				− 0.002
				(0.006)
Frequency				0.001
				(0.003)
Diversity				0.047
				(0.068)
Age	− 0.528*	− 0.407	0.007	0.063*
	(0.316)	(0.435)	(0.020)	(0.036)
Income	0.387*	0.158	0.007	0.037**
	(0.220)	(0.269)	(0.012)	(0.017)
Married^d^	0.799	2.988***	0.116***	− 0.067
	(0.650)	(0.793)	(0.035)	(0.052)
Number of drinks	0.103	0.095	− 0.005	0.003
	(0.145)	(0.177)	(0.008)	(0.011)
Smoking cigarette	− 0.142	− 0.249	0.091*	− 0.087
	(0.863)	(1.052)	(0.047)	(0.068)
Constant	7.916***	12.287***	0.560***	− 0.028
	(0.773)	(0.943)	(0.042)	(0.082)
Observations	1008	1008	1008	1008

This table presents the fixed-effect Instrumental Variable estimation results of parallel mediation equations corresponding to Fig. 2 with anxiety as the outcome variable. Instrument is a dummy variable with value 1 if individual is older than 65 years old, and 0 otherwise. Columns 2-4 present the FE-IV estimation of social network variables on the retirement, corresponding to the paths in Fig. 2

Robust individuals-clustered standard errors are in parenthesis

^a^Size refers to the number of members in the individual’s social network

^b^ Frequency refers to the frequency of contacts with members of one’s social network

^c^ Diversity refers to diversity in the types of relationships in individual’s social network that is measured by Index of Qualitative Variation (IQV)

^d^ Married is a dummy variable that takes value of 1 if individual is married, and 0 otherwise. The sample is limited to pre-retirement healthy individuals in all health outcomes in this study

The significance level is defined as follows: *** significant at the 1% level; ** significant at the 5% level; * significant at the 10% level

## Footnotes

1 Social Security Administration Annual Report.

2 Dave et al.'s study (2008) is the only study on US data which considers not working as the definition of retirement (using Health and Retirement Study dataset) and exploits the panel nature of data.

3 The NSHAP has not been exploited in the literature of retirement and health studies.

4 There are many other studies based on the evidence of other countries such as Korea, Japan, Canada, and European countries.

5 Recently, several studies attempt to investigate how social network impacts retirement decision making (van den Berg et al. 2010; Harkonmäki et al. 2006). Findings show that social support can buffer the effect of main factors of early retirement decision such as poor health, low job satisfaction, and work pressure (van den Berg et al. 2010).

6 We use sample weights for all the analyses.

7 The answer scores to positive notion questions (e.g., “How often did you feel happy in the past week?”) are reversed to be consistent in measuring depression.

8 Anxiety questions are asked in the leave-behind questionnaire, and around 2600 respondents have answered these questions in each wave. Therefore, the subsample for investigating the anxiety impact has the lowest sample size.

9 Respondents were asked: “Are you retired?” possible answers: Yes, No, refused. “Are you currently working?” possible answers: Yes, No, refused. A respondent is considered as retired if the answer to the former question is yes and the latter question is no.

10 Age is entered to the models linearly.

11 CESD score lower than 16 implies no depression symptoms exist (Radloff 1977). Also, anxiety scores below 8 indicates no anxiety is diagnosed (Zigmond and Snaith 1983)

12 This is according to our definition of retirement.

13 For instance, including individuals who retired after 65 years old due to health problems results in overestimation of retirement’s impact on health.

14 The mediation analysis models first introduced by Baron and Kenny (1986) and developed further with less restrictive assumptions in later years, i.e., Zhao et al. (2010). We follow the revised mediation analysis by Zhao et al. (2010).

15 The direct effect of retirement on health cannot be consistently estimated by IV without also instrumenting social networks, since controlling for social networks necessarily invalidates the instrument. To address the possible endogeneity of retirement w.r.t. social network, we have performed exogeneity test. The *p*-value of the exogeneity test (H0: retirement is exogenous) w.r.t size, frequency of contacts, and diversity of social network is 0.001, 0.21, and 0.19, respectively. As the test results show, we have failed to reject the exogeneity of retirement w.r.t. frequency of contacts and diversity of social networks. However, the null hypothesis regarding the exogeneity of retirement w.r.t. the size of the social network variable was rejected. That is, there might be a potential endogeneity of retirement w.r.t. the size of social network. To investigate the impacts of the possible endogeneity of retirement w.r.t. the size of social network on our results, we have followed the causal mediation analysis framework developed by Dippel et al. (2019) to estimate the mediatory effect of social network size under the assumption that both treatment and mediator are endogenous. This method needs a further assumption that the unobserved confounding variables that cause the retirement and the social network size are independent of the confounders that cause the social network size and health (called partially confounding condition). In our study, this assumption is likely to hold because our sample is pre-retirement healthy older adults. Using this method, the mediatory impact of social network remains the same with larger magnitude. The results are available upon request.

16 We mainly used the XTIVREG command in STATA.

17 Table 5 in Appendix presents the first-stage estimation results.

18 In Appendix, Table 6 shows a significant impact of social network on health outcomes using OLS estimation, which ignores the existence of time-invariant confounding, i.e., unobservable time-invariant confounding are the factors affecting both social network and health.
